# Histaminergic Mechanisms for Modulation of Memory Systems

**DOI:** 10.1155/2011/328602

**Published:** 2011-08-18

**Authors:** Cristiano André Köhler, Weber Cláudio da Silva, Fernando Benetti, Juliana Sartori Bonini

**Affiliations:** ^1^Programa de Pós-Graduação em Ciências Médicas, Faculdade de Medicina, Universidade Federal do Rio Grande do Sul, Ramiro Barcelos 2400, 90035-903 Porto Alegre, RS, Brazil; ^2^Centro de Memória, Instituto do Cérebro, Pontifícia Universidade Católica do Rio Grande do Sul, Ipiranga 6690, 90610-000 Porto Alegre, RS, Brazil; ^3^Departamento de Farmácia, Universidade Estadual do Centro Oeste, Simeão Camargo de Sá 03, 85040-080 Guarapuava, PR, Brazil

## Abstract

Encoding for several memory types requires neural changes and the activity of distinct regions across the brain. These areas receive broad projections originating in nuclei located in the brainstem which are capable of modulating the activity of a particular area. The histaminergic system is one of the major modulatory systems, and it regulates basic homeostatic and higher functions including arousal, circadian, and feeding rhythms, and cognition. There is now evidence that histamine can modulate learning in different types of behavioral tasks, but the exact course of modulation and its mechanisms are controversial. In the present paper we review the involvement of the histaminergic system and the effects histaminergic receptor agonists/antagonists have on the performance of tasks associated with the main memory types as well as evidence provided by studies with knockout models. Thus, we aim to summarize the possible effects histamine has on modulation of circuits involved in memory formation.

## 1. Introduction

The initial step in the formation of a new memory is the acquisition of information related to that particular trace, coming from external stimuli (sensory input arising from subject-environment interaction) or from internal representations (cognition and emotion) [1–5]. This acquisition phase is referred to as learning. After the subject learns, information must be stored either for a brief or a long period of time. While that information remains stored, it can be accessed by the process known as retrieval. Memory, thus comprises the processes responsible for retention and retrieval of learned experience [5, 6]. Short-term memory retention (defined in minutes or hours) may be converted to long-term retention (defined in days, weeks, or even years) by a specific sequence of events called consolidation which starts immediately after the acquisition phase [1, 3–7]. Finally, as time passes, even the most consolidated memories may disappear, a process called forgetting [8].

Not all information reaching the central nervous system (CNS) is stored. Most inputs do not pass the acquisition phase, because they are filtered out by attentional and emotional mechanisms [1, 7–9]. Only a few inputs selected for acquisition and retention are consolidated in long-term memories, and many of the latter are forgotten. Only memories relevant for cognition, emotionally more salient, more focused by attention or associated to stronger sensory input will actually persist. In this context, forgetting may also be seen as a filter, since it only affects memories previously selected to persist by emotional and attentional filters. Thus, the brain systems responsible for learning and memory have a mechanism for preventing information overload [5, 8, 10].

Most CNS functions may be modulated so as to be activated or deactivated, accelerated or slowed down, and enhanced or diminished, but the exact course of action is determined by the needs relevant for a particular moment [10]. Memory is no exception to this rule and can thus be modulated by experiences occurring about the time when it is learned, consolidated, or retrieved [7]. The major modulatory systems are composed of diffusely spread fiber bundles that reach a broad area in the CNS. These fibers originate from nuclei in the brainstem, diencephalon, and basal forebrain. They act by means of several neurotransmitters, including acetylcholine, noradrenaline, dopamine, serotonin, and histamine [7, 11–13].

Histamine is involved in the control of several behavioral and neurobiological functions such as the sleep-wake cycle, water intake, motor activity, and nociception [13, 14]. Histamine is known to decrease calcium-dependent membrane conductance in the hippocampus [15], to increase neuronal excitability [16], and control high-frequency oscillations [17, 18], and it also facilitates NMDA glutamatergic receptor-mediated responses [19]. However, the part histaminergic circuits play in mnemonic systems is complex. Histamine seems to have different effects in distinct brain regions and may have modulatory effects that differ according to memory type. The evidence indicating the exact role of histamine in learning processes and memory consolidation is still controversial. In particular, the action of the receptor subtypes and how they affect key circuits related to a specific memory system is not well understood.

Here, we will first present a short review of the anatomy, biochemistry, and physiology of the histaminergic system and synaptic plasticity. Then, we will summarize pharmacological studies and other evidence that aims to identify the possible role of histamine in the modulation of brain regions pertaining to circuits involved in processing several types of memories.

## 2. Anatomy of the Histaminergic System

**Figure 1:**
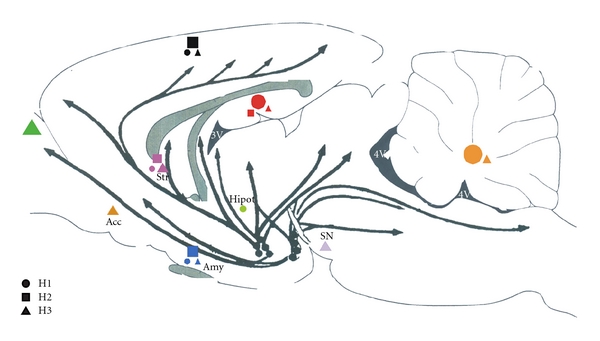
Main histaminergic projections and histamine receptor distribution in the rat brain. The shapes mark the main sites where each receptor subtype is located. In areas that have more than one receptor subtype, the shape with the biggest size indicates which is predominant. Acc: nucleus accumbens; Amy: amygdala, Hipot: hypothalamus, Str: striatum, SN: substantia nigra.

Here, we highlight the key aspects of the anatomy of the histaminergic system. For more details, the reader is encouraged to see [20–23].  Figure 1 presents the main projections of histaminergic neurons in the rat brain.

In vertebrates, neurons that produce histamine are located in the tuberomammillary nucleus (TMN), which is part of the posterior hypothalamus [14, 21]. The main afferent projections to the TMN come from the infralimbic cortex, lateral septum, preoptical nucleus, and neuronal groups in the brainstem [23, 24]. 

The final target of histaminergic fibers varies slightly across species, but they reach almost the entire CNS. There are moderate-to-dense histaminergic nerve terminals in the cerebral cortex, amygdala, substantia nigra, striatum, hippocampus, and thalamus [20–22]. As a result of these broad projections, histamine receptors are distributed widely in the CNS.

## 3. Histamine Metabolism and Neuronal Turnover

In neurons, histamine is synthesized from the amino acid L-histidine, which is transported from the extracellular into the intracellular space by the L-aminoacid transporter located in the cell membrane. Once in the cytoplasm, L-histidine is decarboxylated by the enzyme histidine decarboxylase, with histamine as the end product of the reaction. This newly synthesized histamine is transported into synaptic vesicles by the vesicular monoamine transporter 2 (VMAT-2). Action potentials that reach the axonal endings trigger the fusion of the vesicles with the presynaptic membrane, releasing histamine in the synaptic cleft [13, 14]. 

In the extracellular space, histamine is methylated by enzyme histamine methyltransferase in the postsynaptic membrane and glial cells. The end product of this methylation reaction is telemethylhistamine, a metabolite that has no histaminergic activity. Since this amine does not have a high-affinity reuptake mechanism, methylation is the main mechanism for inactivation of released histamine [13, 14]. The turnover rate of neuronal histamine is high, and its half-life (which is about 30 minutes in basal conditions) can change rapidly in response to neuronal activity. Turnover rate increases when subjects experience stressful events such as forced immobilization or a foot shock [21].

The control of histamine synthesis and release is mediated by H3 type autoreceptors that are located in the soma and axonal endings of the histaminergic neurons [25]. Histamine release may also be regulated by inhibitory M1 muscarinic receptors [25–27], *α*2 adrenergic receptors [26, 28, 29], 5-HT1A serotonergic receptors [30], **κ** opioid receptors [26, 31] and galanin receptors [26], or facilitatory *μ* opioid receptors [32]. In vivo experiments have shown nitric oxide to inhibit histamine release in the hypothalamus [33].

The circadian rhythm of histamine release coincides with changes in the firing pattern of histaminergic neurons occurring during the sleep-wake cycle [14, 33]. TMN histaminergic neurons resemble a pacemaker activity pattern: they fire action potentials at a slow frequency (<3 Hz). Electrophysiological recordings of these neurons in cats showed their activity increased during wake time, and decreased or was completely absent during sleep [14, 34, 35]. However, histamine release can follow a faster ultradian rhythm correlated to the delta and theta bands in electroencephalographic recordings [14, 36].

## 4. Histaminergic Receptors

In the nervous system of vertebrates most histaminergic axonal endings (varicosities) are not in close contact with postsynaptic sites [37]. This causes histamine to have a diffuse action pattern which is similar to other biogenic amines [14].

To date, histamine effects are known to be mediated by four distinct types of receptors (H1 to H4) coupled to G proteins and several associated second messengers.  Figure 1 shows their distribution across the rat brain. H1, H2, and H3 receptor types are expressed mainly in nervous tissues, and H4 type in peripheral tissues like bone marrow and leukocytes. Generally, H1 and H2 receptors excite or potentiate excitatory impulses [14, 37–39], while H3 activation mediates autoinhibition of TMN neurons and inhibits the synthesis and release of histamine or other neurotransmitters such as glutamate, acetylcholine, and noradrenaline [14, 40, 41].

The H1 receptor is a protein with 486–491 amino acid residues and is encoded by a gene without introns [14, 42]. It is coupled to the activation of the G_q/11_ protein, which activates phospholipase C (PLC) [13]. Thus, the intracellular effects of H1 receptor activation are mediated by lipid second messengers related to intracellular calcium stores. Downstream signaling pathways are involved in synaptic plasticity. This receptor has been identified in the cortex, hippocampus, amygdala, hypothalamus, thalamus, striatum, and cerebellum [43].

The H2 receptor is also encoded by a gene without introns and is a protein with 358-359 amino acid residues [14, 44, 45]. It is coupled to the G_s_  protein, which stimulates adenylate cyclase and, subsequently, protein kinase A (PKA) [13]. This receptor is mainly found in the basal ganglia, amygdala, hippocampus, and cortex. H2 receptor activation also provokes downstream signaling cascades, which are able to mediate functional and structural changes in synapses, as in the case of the PKA pathway [46].

The H3 receptor has several isoforms [47, 48] and is a protein with 326–445 amino acid residues. The isoforms originate in the expression of a single gene through the alternative processing of mRNA (splicing). The intracellular effects of this receptor start with the activation of the inhibitory G_i_/G_o_ protein and the subsequent inhibition of adenylate cyclase [13]. The H3 receptor is found in histaminergic neurons as an autoreceptor, and its intracellular inhibitory actions control histamine synthesis and release [25, 49–51]. This receptor subtype can also regulate the release of acetylcholine, dopamine, GABA, glutamate, noradrenaline, 5-HT, and tachykinins by means of this presynaptic mechanism [14, 40, 41, 52–54]. The H3 receptor also participates in the activation of the MAPK signaling pathway [48]. Its mRNA is found in several types of neurons and has been identified in rats mainly the cerebral cortex, the hippocampus, tenia tecta, nucleus accumbens, caudate putamen, thalamus, amygdala, cerebellum, vestibular nuclei, and some hypothalamic nuclei [55]. The actual location of the receptor protein may be very distant from the cell bodies that express mRNA, and the major brain sites where binding activity takes place are the cerebral cortex, amygdala, nucleus accumbens, caudate putamen, globus pallidus, and substantia nigra [55].

## 5. Histamine and Synaptic Plasticity

Long-term potentiation (LTP) is considered the main demonstration of synaptic plasticity. It was first described in the hippocampus, a brain region directly involved in the formation of several memory types [10, 56–58]. It consists of the lasting enhancement of the postsynaptic response following a high-frequency afferent stimulation. This is seen as an increase in the excitatory postsynaptic potential evoked by a single stimulation pulse when compared to the response evoked before the high-frequency stimulation [57]. There is evidence that this form of synaptic enhancement is also present in other regions of the cerebral cortex, especially during early developmental stages [59]. LTP has several characteristics that make it a strong candidate for the cellular mechanism responsible for long-term memory storage (see [59] for a more detailed review about this matter).

Among the several mechanisms that are jointly responsible for LTP, the activation of the glutamatergic NMDA receptor plays a central role in both the induction and maintenance of most LTP types [59]. The calcium influx in the postsynaptic dendritic spine leads to the activation of several signaling cascades, notably the calcium/calmodulin-dependent protein kinase II (CaMKII), the protein kinase A (PKA), the protein kinase C (PKC), and the mitogen-activated protein kinase (MAPK). Their final course of action is the activation of transcription factors like CREB, which control the synthesis of the proteins required for the maintenance of the increased postsynaptic response [59–62].

This process may be modulated by histamine [63]. LTP was facilitated by histamine in the CA1 area of rat hippocampal slices, since it could be induced with a weak tetanic stimulation when histamine was added to the bath, and this effect persisted even in the presence of H1 and H2 receptor antagonists. It was then demonstrated that histamine or its major metabolite, 1-methylhistamine [64], may enhance NMDA induced response and subsequently hippocampal LTP by means of direct channel activation by binding to the polyamine site [14, 65, 66].

Regarding the effects of histamine on LTP, H1 receptor activation has been shown to reduce the blockage of the NMDA receptor channel mediated by the ion Mg^2+^, by activating PKC, which leads to increased activation of the glutamatergic receptor [67]. In addition, as already pointed out, histamine can also directly activate the NMDA receptor by binding to the polyamine modulatory site present in this receptor [19, 68]. Binding is pH sensitive and is restricted to the NR1/NR2B subunits of the NMDA receptor [64]. Furthermore, H1 receptor activation leads to the synthesis of postsynaptic retrograde messengers, such as nitric oxide and arachidonic acid, which might be responsible for presynaptic modifications that occur after LTP induction [14, 41]. As an example of the possible involvement of this receptor in LTP, H1 receptor knockout mice showed impaired LTP induction in the CA1 region of the hippocampus [68]. 

As mentioned above, the H2 receptor is a potent stimulator of the cAMP signaling pathway, which is necessary for the late phase of NMDA receptor-dependent LTP [69] and for LTP expression in giant hippocampal mossy fiber synapses [70]. H2 receptors can also increase ionic currents generated by the activation of the NMDA receptor, through blockage of calcium-dependent potassium channels [71], and possibly by mediating the phosphorylation of the NMDA receptor itself by PKA [72]. LTP induction in the hippocampus was also impaired in H2 receptor knockout mice [68].

The effects might change across different brain regions. H3 receptors can reduce the release of several neurotransmitters, including glutamate, which might affect synaptic plasticity in hippocampus and striatum [40, 73]. In dentate gyrus, for instance, H3 receptor activation hinders synaptic transmission and reduces paired-pulsed facilitation, which is a short-term form of synaptic plasticity [74]. In the CA3 region of the hippocampus, histamine promotes synchronized bursts of action potentials [75], an activity pattern that is known to be a physiological stimulus for the occurrence of LTP in the CA1 region [76]. In the CA1 region, when Ca^2+^ levels are low and Mg^2+^ levels are high, histamine causes a lasting potentiation of neuronal excitability, which is mediated mainly by H2 receptor activation and the PKA signaling pathway, with H1 and NMDA receptors having a modulatory role in that downstream cascade. 

Thus, histamine can enhance LTP through at least three different mechanisms: H1 receptors favor LTP by increasing intracellular levels of Ca^2+^ and subsequent PKC activation, both required for LTP induction, the effects of the H2 receptors are mediated by cAMP/PKA signaling pathway activation, which is involved in the maintenance of LTP, and finally, the NMDA receptor can be directly activated by histamine binding to the polyamine site, which can modulate both LTP induction and maintenance [14, 65, 66].

## 6. Histamine and Memory

### 6.1. Systemic Administration of Histamine and Memory Performance

The first approach to elucidate the possible role of histamine on memory systems consisted in the systemic administration of several drugs that mimic or counteract the action of histamine and its receptors. These studies are summarized in Table 1. 

#### 6.1.1. Memory Impairment

There is a considerable body of work on the role of histamine in models of memory impairment induced by systemic administration of antagonists of the neurotransmitters acetylcholine and glutamate, most notably using the muscarinic receptor antagonist scopolamine or the NMDA receptor antagonist MK-801. The reversal of memory deficit produced by systemic administration of histamine in these models of memory impairment was evaluated in several behavioral tasks (see Table 1 for references). 

The evidence suggests that this reversal comes about as a result of the interplay between the histaminergic and cholinergic systems. It is known that both cholinergic and gabaergic projections of the septum to the hippocampus are important in generating hippocampal theta rhythm [100, 101]. It has been demonstrated that histamine can activate gabaergic neurons in the septohippocampal pathway, by binding to the H1 and H2 receptors [102]. Furthermore, electrical stimulation of TMN has been shown to increase acetylcholine (ACh) release in the hippocampus [103], and intraseptal infusions of histamine also enhance hippocampal ACh release [104]. Thus, histamine can regulate the hippocampal electrical activity and induce ACh release in the hippocampus in physiological states. The improvement seen in scopolamine-induced deficits might be mediated by such modulation of the septohippocampal pathway.

#### 6.1.2. Physiological Context

Systemic studies that investigate the impact histamine has on memory in a physiological context show the effect to differ with respect to the behavioral paradigm, and thus the memory type, and most studies, deals with the direct manipulation of a single histamine receptor (Table 1). 

In aversive tasks like inhibitory avoidance, the systemic administration of an H3 receptor antagonist improves performance (see Table 1 for references). These studies have used ligands with varying degrees of potency, bioavailability, and affinity for studying species receptor [105]. While some studies used classical ligands now classified as inverse agonists (like thioperamide and clobenpropit, which have nonspecific actions, notably on the 5-HT_3_ serotonergic receptor and *α*_2C_ adrenergic receptor [105]), studies employing ligands with a different chemical structure and with more selectivity and affinity to the H3 receptor showed similar memory enhancing effects. By contrast, the administration of an H3 receptor agonist impairs performance [96], and both non-specific ligands like RAMH (also active on noradrenergic receptors) and imetit (also active on serotonergic receptors) and the more selective ligand immepip produced similar memory impairment effects. Together, these data highlight the possible role of H3-mediated modulation on aversive learning. 

On the other hand, the administration of an H1 receptor antagonist has produced different results for aversive memory tasks [97, 98]. It must be noted, though, that there is an age difference in the experimental subjects. The expression pattern of the histamine receptor subtypes alters with age in rodents. Since the H1 receptor has the most widespread changes in density [106], this might account for the opposite effect. Finally, the alkylamine chlorpheniramine used in the study also has nonspecific actions, notably the inhibition of noradrenaline and serotonin reuptake, which could also account for the reported effects [107, 108]. Although there are some differences between passive and active avoidance tasks regarding learning (active avoidance involves multitrial learning task, while passive avoidance is singletrial, the latter being more suitable to pharmacologically dissect the role of certain receptors or signaling pathways), the only study that evaluated first generation and the more selective second generation H1 blockers using active avoidance showed that those nonspecific effects might indeed play a role in the evaluation of the action of H1 receptors on aversive memory: although all the compounds impaired learning, the extent of the impairment seems to be greater for the nonspecific compounds [98].

The results of systemic studies are contradictory regarding the effect the histaminergic system has on spatial memory tasks such as the water maze (Table 1). The administration of a histamine precursor did not produce any effects, while the administration of an H3 receptor agonist improved performance [99]. This may be explained by the fact that the H3 receptor is also found on neurons that release other neurotransmitters, thus improvement could be mediated by the regulation of other systems involved in spatial learning, such as the dopaminergic system [109, 110], and not only by increasing the synaptic availability of histamine. Another explanation would be that the H3 agonist RAMH ligand is not specific to histamine and may also have affinity for other aminergic receptors [105].

### 6.2. Transgenic Models

Recent studies have used transgenic models to try to identify the role of histamine and each receptor subtype on memory processes. These studies are summarized in Table 2.

There is evidence that the lack of the histamine decarboxylase enzyme produces changes in the performance of some memory tasks although the course might also differ with respect to memory type. In the water maze task, spatial memory has been shown to be both hindered and improved in histamine decarboxylase knockout mice (Hdc −/− mice) [111, 112] though these differences may be influenced by the gender base used in the models. Thus, although the evidence provided by the transgenic models shows that histamine participates in the modulation of at least some memory systems, the exact course of its actions remains unclear. 

Knockout models for H1 and H2 receptors, on the other hand, have shed more light on the physiological role of histamine in learning. They act negatively on the learning of aversive tasks but facilitate spatial and discriminative ones. Interestingly, the only study that addresses the role of the three histamine receptors in a Barnes-maze task showed that the lack of an H3 receptor improves memory. The absence of presynaptic autoinhibitory receptors might potentiate the action of histamine in postsynaptic H1 and H2 receptors, which also modulated the memory associated with this particular task (see Table 2 for references).

An important factor in the interpretation of studies with knockout models is that the inactivation of the genes of interest is not restricted to an area or specific part of the circuits involved, and thus, these effects are likely to reflect the overall action of histamine or its receptors. Maybe studies using temporally and spatially restricted knockout models could clarify the role of histamine in each brain site leading to a better understanding regarding how it modulates circuits responsible for processing different memory types.

### 6.3. Intracerebroventricular (ICV) Administration of Histamine and Memory Performance

The administration of histamine or its receptor agonists/antagonists in the ventricular system is a more convenient way to study the role of histamine in the CNS than peripheral administration, since some ligands might be more prone to cross the blood-brain barrier, and there is less potential for peripheral side effects. In Table 3, we have summarized the studies that employ this delivery route in attempting to address the role of the histaminergic system. 

Histamine had facilitatory actions in social learning, since its ICV infusion improved, while the inhibition of its synthesis hindered the performance. Also, pharmacological stimulation with the potent and selective H3 agonist immepip led to impaired performance in learning the task, while the use of the inverse agonist thioperamide facilitated it. The role of the other receptor types is not addressed, and thus, the H3 receptor response and histamine effect might be due to the regulation of other neurotransmitter systems. 

Conversely, the findings of studies that used tasks based on learning an aversive memory are controversial. Studies that employed a passive avoidance task showed either improvement or impairment of the task following ICV administration of histamine [120–124], but the doses administered varied: 1–10 ng/rat of histamine improved performance, while 20 *μ*g/rat impaired it. EC_50_ of the H3 receptors are at least one order of magnitude lower than H1 and H2 receptors [13], and thus, the low concentration might be causing a mainly H3-based response, thus acting negatively on the histaminergic system, which would explain the reported improvement in aversive memory. Since H1 and H2 receptor blockage also improved the memory for the same task, this would corroborate the inhibitory actions of histamine in aversive learning [122–124]. 

The findings of two studies employing the active avoidance paradigm were also contradictory [127, 135], but the study that showed memory improvement was found with the use of aged rats, which might account for the reported contradictory effects. Finally, there is controversy regarding the role of the H1 receptor in the active avoidance task, since it was shown that this could either inhibit or facilitate performance in active avoidance tasks [127, 132].

The ICV administration of compounds that act on the histaminergic system is less studied in spatial memory and object recognition or discriminative memory types. Histamine reverses the impairment induced by NMDA antagonists in a radial maze task [130]. The activation of both glycine and polyamine sites can act synergistically in memory function [136], and thus, histamine might compensate the deficit through direct action on the NMDA receptor. However, in a physiological context, the blockage of histamine synthesis [131] or lack of the gene that encodes the H1 receptor impaired performance in the radial maze [117], while H1 receptor inactivation improved the memory in the water-maze task [134]. Methodological particularities of these tasks that rely on spatial memory might explain this differential effect. H1 receptor activation facilitated learning the object recognition task, and this is in agreement with studies that used knockout models (Table 2). The brain areas involved are slightly different for spatial and discriminative tasks, with spatial memory depending mainly on the hippocampus, while discriminative memory relies on other cortical areas, such as the perirhinal cortex [137–139]. Thus, the different roles the H1 receptor seems to have in spatial and recognition memory might rely on those distinct anatomic requirements, since the density of H1 receptors is different in the cortex and the hippocampus.

### 6.4. Administration of Histamine into the Hippocampus and Memory Performance

The hippocampal formation is composed of three subregions: the Ammon's horn, the dentate gyrus, and the subiculum. The Ammon's horn is further divided into the subfields CA1–CA4. These subregions are connected to form a closed-loop circuit with the adjacent entorhinal cortex (EC). Neurons from the layer II of the EC project to the dentate gyrus and the CA3 subfield, while neurons from layer III project directly to CA1. The projections from the EC are known as the perforant pathway. Neurons from the DG project to the CA3 subfield, through the mossy fiber pathway. CA3 neurons make synapse with neurons from the CA1 region through the Schaffer collateral pathway. Finally, to close the loop, the CA1 region neurons project to the layer V of the entorhinal cortex [148, 149].

The superficial entorhinal layers receive input from other cortical areas and the olfactory bulb. The CA1 area is the main output of the hippocampus, which is directed mainly to the EC, which act as a hub to other cortical areas. The hippocampus can also project to subcortical areas, mainly through subiculum. Among the subcortical input to the hippocampus are projections from the septum, amygdala, thalamic nuclei, diagonal band of Broca, the basal nucleus of Meynert, the tuberomammillary nucleus, the ventral tegmental area, the raphe nuclei, and the locus coeruleus. Finally, the connectivity of the hippocampus to other structures differs with respect to its dorsoventral axis [148–150].

The hippocampus is central in spatial memory tasks [56], and the NMDA receptor plays an important role in this type of learning [151]. The impairment in radial maze learning induced by the NMDA receptor antagonist MK-801 could be reversed by the direct administration of histamine in the hippocampus [78]. Since the blockage of the H3 receptor in this structure had a similar effect and the reversal was hindered by the administration of an H1 receptor antagonist [78], the observed improvement might be due to the increased synaptic availability of histamine and its binding to postsynaptic H1 receptors [78]. The modulation of the excitability of hippocampal cells through the H1 and H2 receptors [13] and the direct activation of the NMDA receptor through the polyamine site [65, 66] are possible mechanisms to mediate the observed effects.

The inhibition of the septum or the administration of scopolamine cause memory deficits that are reversed by histamine or the H3 receptor antagonist clobenpropit (Table 4). Together with the mechanisms described for the modulation of the septohippocampal pathway [102], histamine seems to locally regulate ACh release in the hippocampus.

The hippocampus is also required for learning aversive tasks like inhibitory avoidance and contextual fear conditioning [10, 152]. There is a possible anatomical dissociation within this region regarding the effects of histamine on aversive tasks, since in the dorsal hippocampus histamine seems to facilitate both fear conditioning and passive avoidance through the H2 receptor, while in the ventral area, it has inhibitory effects on active avoidance through the H1 receptor (Table 4). The extent to which the methodological peculiarities of each aversive paradigm play a role in this dissociation remains unclear. In addition, there is evidence that the hippocampus has a functional segmentation in its dorsoventral axis, with spatially distinct molecular domains and subdomains [150]. This might lead to differences in receptor density or downstream signaling pathways that together could account for the observed results.

Thus, the mechanisms that mediate the effects seen in this area may result from the interplay of histamine with cholinergic and other aminergic systems, direct activation of the glutamatergic system through the NMDA receptor, or by cellular downstream cascades activated from a particular receptor, with a possible overall combination of one or more mechanisms [13, 153] that altogether could account for discrepancies and differential involvement of histamine in a particular memory task.

### 6.5. Administration of Histamine into the Amygdala and Memory Performance

The amygdala is a complex mass of gray matter that comprises multiple and distinct subnuclei and is richly connected to nearby cortical areas that constitute the amygdala circuitry. The amygdala contains three functional subdivisions, each one having a unique set of connections with other regions of the brain. The medial group of subnuclei has extensive connections with the olfactory bulb and the olfactory cortex. The basolateral group, which is especially large in humans, has major connections with the cerebral cortex, especially the orbital and medial prefrontal cortex. The central and anterior group of nuclei is characterized by connections with the brainstem and hypothalamus and with visceral sensory structures, such as the nucleus of the solitary tract. For more details about the general organization of the amygdala anatomy circuitry, see [157]. 

Previous studies have clearly identified the amygdala as a key brain structure for acquisition and storage of several memory types, first among them, fear memory. Classical fear conditioning is a powerful behavioral paradigm that has been widely studied in amygdala nuclei and mainly in the basolateral amygdala nucleus (BLA), which have been shown to participate in the learning and memory consolidation mechanism [158]. These findings are consistently supported by a large number of studies using different experimental paradigms and measures of aversive memory [158, 159]. In addition, the amygdala also modulates fear-related learning in the other brain structures, such as the cortex and the hippocampus [160].

The studies that investigate the modulatory effects of histamine in the BLA are summarized in Table 5. Electrophysiological studies in rat brain slices revealed that histamine can have bidirectional effects on excitatory synaptic transmission in BLA depending on the blockage of H3 receptors: the excitatory postsynaptic potential was depressed in the presence of histamine alone but increased when the H3 receptor antagonist thioperamide was added to the preparation [161]. Behavioral models, however, are better suited to study such effects, since the slice preparation lacks afferent and efferent projections. Few behavioral studies have evaluated the role of histamine in amygdala.

Evidence using the fear conditioning task with pharmacological agonists/antagonists showed that blockage of the H3 receptors has inhibitory actions, while their activation improves the expression of fear memory. It has been shown that the modulation of ACh release in amygdala through the H3 receptor participates in this modulation, since rats that received the infusion of H3 receptor antagonists in the BLA at similar concentrations to those that affected fear memory had a significant reduction in the spontaneous release of ACh [154]. New approaches using transgenic models agree with the inhibitory role for histamine in fear memory, since the lack of H1 and H2 receptors improves memory for this task, and mice with reduced levels of histamine due to lack of histamine decarboxylase also have improved performance although the inactivation was not restricted to the BLA (Table 2). Along with pharmacological data, this suggests that these inhibitory effects might also be mediated by the H1 and H2 receptors.

## 7. Conclusion

Together, these studies suggest that the manipulation of the central histaminergic system affects behavioral responses during several learning and memory processes. However, the results are often contradictory and do not establish whether the overall action of histamine on the memory system is either facilitatory or inhibitory. Methodological issues, such as time and the methods used in the administration of the compounds under scrutiny as well as the parameters related to the behavioral task under evaluation need to be taken into consideration. Furthermore, in view of the opposite effects that the activation of the different histamine-receptor subtypes has on memory due to the variability of cellular action, the final course of action of histamine in a network is not predictable.

##  Conflict of Interests

The authors declare that they have no conflict of interest.

## Figures and Tables

**Table 1:** Systemic administration of drugs that act on the histaminergic system and memory performance.

Species	Histaminergic modulator delivery route	Behavioral task	Histaminergic modulator	Lesion or pharmacological induced memory impairment	Effect on memory	Reference(s)
Rat	i.p.	Radial maze	Histidine	scopolamine (intrahippocampal)	↑	[77]
Rat	i.p.	Eight-arm radial maze	Histidine	MK-801 (intrahippocampal)	↑	[78]
Rat	i.p.	Delayed non-matching to position (DNMTP) task	H3 receptor antagonist (JNJ-10181457)	Scopolamine (i.p.)	↑	[79]
Rat	i.p.	Radial maze	Histidine	Muscimol (intraseptal)	↑	[80]
Rat	i.p.	Object recognition	H1 receptor antagonist (ciproxifan)	Scopolamine (i.p.)	↑	[81]
Mouse	i.p.	Object recognition	H3 receptor inverse agonist (BF 2649)	Scopolamine (i.p.)	↑	[82]
Rat	i.p.	Passive avoidance	H3 receptor antagonist (compound 5)	Scopolamine (s.c.)	↑	[83]
Rat	Oral administration	Passive avoidance	H3 receptor antagonist (GSK207040 and GSK334429)	Scopolamine (i.p.)	↑	[84]
Mouse	i.p.	Step-through inhibitory avoidance	H3 receptor antagonist (thioperamide)	scopolamine or dizocilpine (i.p.)	↑	[85]
Mouse	i.p.	Step-through passive avoidance	H3 receptor antagonist (clobenpropit, thioperamide)	scopolamine (i.p.)	↑	[86, 87]
Mouse	i.p.	Plus maze	Histidine	Scopolamine (i.p.)	↑	[88]
Mouse	i.p.	Plus maze	H3 receptor antagonist (thioperamide)	Scopolamine (i.p.)	↑	[89]
Mouse	i.p.	Plus maze	H3 receptor antagonist (FUB 181 [3-(4-chlorophenyl)propyl-3-(1H-imidazol-4-yl)propyl ether])	scopolamine (i.p.)	↑	[90, 91]
Rat	s.c.	Inhibitory avoidance	H3 receptor antagonists (GT-2331, ciproxifan, A-304121, A-317920; ABT-239)		↑	[92–94]
Mouse	i.p.	Step-through inhibitory avoidance	H3 receptor antagonist (thioperamide)		↑	[85]
Mouse	i.p.	Passive avoidance	H3 receptor antagonist (thioperamide)		↑	[95]
Rats	i.p.	Object recognition Passive avoidance	H3 receptor agonists (RAMH, imetit, immepip)		↓	[96]
Rat	Oral administration.	Active avoidance	H1 receptor antagonists (diphenhydramine, pyrilamine, promethazine and chlorpheniramine)		↓	[97]
Rat	i.p.	Inhibitory avoidance	H1 receptor antagonist (chlorpheniramine)		↑	[98]
Rat	i.p.	Water maze	Histidine		no effect	[99]
Rat	i.p.	Water maze	H3 receptor agonist, (RAMH)		↑	[99]
Rat	i.p. or oral administration	Social memory	H3 receptor antagonists (ABT-239)		↑	[94]

RAMH: (R)-alpha-methylhistamine (H3 receptor agonist); i.p.. intraperitoneal; s.c*.:* subcutaneous.

**Table 2:** Knockout mice models and memory performance.

Behavioral task	Knockout type	Effect on memory	Reference(s)
Water maze*	Hdc −/−	↓	[111]
Water maze	Hdc −/−	↑	[112]
Object recognition*	Hdc −/−	Unaffected	[111]
Nonreinforced episodic object memory	Hdc −/−	↓	[112]
Contextual fear conditioning	Hdc −/−	↑	[113]
One-trial passive avoidance	Hdc −/−	↑	[111]
Open field (habituation)	Hdc −/−	Unaffected	[114]
Inhibitory avoidance	KO H1−/−	Unaffected	[115]
Conditioned place preference	KO H1−/−	↓	[116]
Radial-maze task	KO H1−/−	↓	[117]
Auditory and contextual fear conditioning	KO H1−/−	↑	[68]
Auditory and contextual fear conditioning	KO H2−/−	↑	[68]
Object recognition	KO H1−/−	↓	[68]
Object recognition	KO H2−/−	↓	[68]
Barnes maze	KO H1−/−	↓	[68]
Barnes maze	KO H2−/−	↓	[68]
Barnes maze	KO H3−/−	↑	[118]
Motoric long-term memory	KO H1−/−	↓	[119]
Episodic and procedural memory	KO H1−/−	↓	[119]

*This work was done using female mice.

Hdc: histidine decarboxylase.

H1, H2, H3: histaminergic receptor type.

**Table 3:** Intracerebroventricular administration of drugs that act on the histaminergic system and memory performance.

Species	Behavioral task	Histaminergic modulator	Lesion or pharmacological induced memory impairment	Effect on memory	Reference(s)
Rat	Passive avoidance	Histamine		↑	[120, 121]
Rat	Passive avoidance	Histamine		↓	[122–124]
Mouse	Passive avoidance	Histamine	Lithium (i.p.)	↑	[125]
Rat	Active avoidance	Histamine		↑	[126]
Rat	Active avoidance	Histamine		↓	[127]
Rat	Active avoidance	Histamine	Bilateral hippocampectomy (dorsal hippocampus)	↑	[128]
Rat	Active avoidance	Histamine	alpha-FMH (i.p.)	↑	[126]
Rat	Habituation in open field	Histamine		↑	[121]
Rat	Social memory	Histamine		↑	[129]
Rat	Social memory	Histidine		↑	[129]
Rat	Social memory	alpha-FMH		↓	[129]
Rat	Radial maze	Histamine	N-methyl- d-aspartate (NMDA) receptor glycine site antagonist (7-chlorokynurenic acid) i.c.v	↑	[130]
Rat	Radial maze	alpha-FMH		↓	[131]
Rat	Passive avoidance	H1 receptor antagonist (chlorpheniramine)		↑	[122]
Rat	Passive avoidance	H1 receptor antagonist (pyrilamine )		↑	[124]
Rat	Passive avoidance	H2 receptor antagonist (cimetidine)		↑	[123, 124]
Mouse	Passive avoidance	H2 receptor agonist (4-methylhistamine)		↓	[90]
Rat	Active avoidance	H1 receptor agonist (2-methylhistamine and 2-thiazolylethylamine)		↓	[127]
Rat	Step-through active avoidance	H1 receptor agonist (2-methylhistamine)		↑	[132]
Rat	Step-through active avoidance	H1 receptor antagonist (pyrilamine or diphenhydramine)		↓	[132]
Rat	Social memory	H3 receptor agonist (immepip)		↓	[129]
Rat	Social memory	H3 receptor antagonist (thioperamide)		↑	[129]
Rat	Object recognition	H1 receptor agonist [2-(3-(trifluoromethyl)-phenyl)histamine]		↑	[133]
Rat	Water maze	H1 receptor antagonist (chlorpheniramine)		↑	[134]

alpha-FMH: alpha-fluoromethylhistidine (inhibitor of histamine decarboxylase)

i.p. intraperitoneal

i.c.v intracerebroventricular.

**Table 4:** Intrahippocampal administration of drugs that act on the histaminergic system and memory performance.

Species	Hippocampal site	Behavioral task	Histaminergic modulator	Effect on memory	Reference(s)
Rat^1^	Dorsal	Inhibitory avoidance	Histamine	↑	[140]
Rat^2^	Ventral	One-way active avoidance	Histamine	↓	[141–143]
Rat	Dorsal	One-way active avoidance	Histamine	Unaffected	[143]
Rat^2, 3^	Dorsal	Eight-arm radial maze	Histamine	↑	[78]
Rat^4^	Intrahippocampal	Radial maze	Histamine	↑	[80]
Rat	Dorsal	Inhibitory avoidance	H2 receptor agonist (dimaprit)	↑	[140]
Rat^5^	Ventral	Active avoidance	H3 receptor antagonist (clobenpropit)	↑	[144]
Rat	Dorsal	Contextual fear conditioning	H2 receptor agonist (amthamine)	↑	[145]
Rat	Dorsal	Contextual fear conditioning	H3 receptor agonist (RAMH)	↑	[145]
Rat^3^	Dorsal	Eight-arm radial maze	H3 receptor antagonist (clobenpropit)	↑	[146]
Rat	Dorsal	Three-panel runway	H1 receptor antagonist (pyrilamine)	↓	[147]

RAMH: (R)-(−)-alpha-methylhistamine

^1^Effect reverted with concomitant administration of the H2 receptor antagonist ranitidine

^2^Effect reverted with concomitant administration of the H1 receptor antagonist pyrilamine

^3^Impairment induced using intrahippocampal injection of MK-801

^4^Impairment induced using intraseptal injection of muscimol

^5^Impairment induced using intrahippocampal injection of scopolamine.

**Table 5:** Administration of drugs that act on the histaminergic system into the amygdala and memory performance.

Species	Amygdala site	Behavioral task	Histaminergic modulator	Effect on memory	Reference(s)
Rat	Basolateral	Active avoidance	Histamine	↓	[144]
Rat	Basolateral	Contextual fear conditioning	H3 receptor antagonists (ciproxifan, clobenpropit and thioperamide)	↓	[154]
Rat	Basolateral	Contextual fear conditioning	H3 receptor agonists (RAMH or Immepip)	↑	[144, 155]
Rat	Basolateral	Contextual fear conditioning	H3 receptor agonist (proxifan)	↑	[156]

RAMH: (R)-(−)-alpha-methylhistamine.
